# Fatigue and Wear Performance of Autoclave-Processed and Vacuum-Infused Carbon Fibre Reinforced Polymer Gears

**DOI:** 10.3390/polym15071767

**Published:** 2023-04-01

**Authors:** Zoran Bergant, Roman Šturm, Damijan Zorko, Borut Černe

**Affiliations:** Faculty of Mechanical Engineering, University of Ljubljana, Aškerčeva 6, 1000 Ljubljana, Slovenia

**Keywords:** carbon fibre reinforced polymer, gears, fatigue, wear, finite element analysis, autoclave, vacuum infusion

## Abstract

This study focuses on investigating the fatigue and wear behaviour of carbon fibre reinforced polymer (CFRP) gears, which have shown promising potential as lightweight and high-performance alternatives to conventional gears. The gears were fabricated via an autoclave process using an 8-layer composite made of T300 plain weave carbon fabric and ET445 resin and were tested in pair with a 42CrMo4 steel pinion and under nominal tooth bending stress ranging from 60 to 150 MPa. In-situ temperature monitoring was performed, using an infrared camera, and wear rates were regularly assessed. The result of the wear test indicates adhesive wear and three-body abrasion wear mechanisms between the CFRP gears and the steel counterpart. A finite element analysis was performed to examine the in-mesh contact and root stress behaviour of both new and worn gears at various loads and a specified running time. The results point to a substantial divergence from ideal meshing and stress conditions as the wear level is increased. The fatigue results indicated that the CFRP gears exhibited superior performance compared to conventional plastic and composite short-fibrous polymer gears. The described composite gear material was additionally compared with two other composite configurations, including an autoclave-cured T700S plain weave prepreg with DT120 toughened resin and a vacuum-impregnated T300 spread plain weave carbon fabric with LG 900 UV resin. The study found that the use of the T700S-DT120 resulted in additional improvements.

## 1. Introduction

The use of polymer and polymer composite gears has seen a significant rise in recent times, particularly in applications that require low loads where using metal gears may not be cost-efficient. The use of composites is expanding to dynamically loaded applications, including bearings [1,2] and gears. The growing popularity of polymer materials in gear transmissions is attributed to their superior properties, including reduced noise and vibration levels, lower weight, enhanced corrosion resistance, and the ability to operate in lubrication-free environments. The exceptional fatigue and creep resistance of CFRP, compared to other FRPs, has greatly expanded its use in engineering applications where the composite is used as the primary load-bearing component in prestressed applications [3,4,5]. The use of polymer materials in powertrain systems is experiencing exponential growth across a range of fields, including household appliances, hand power tools, medicine, robotics, and beyond. However, a key challenge with currently available lightweight materials is that they typically have significantly lower load capacity compared to steel components, often by a factor of 8–10. To address this issue, CFRP composites have emerged as a class of extremely durable and lightweight materials that can already replace metals in a variety of engineering applications.

For applications with the most severe operating conditions, lightweight CFRP parts are joined with parts made of Fe, Al, and Ti alloys, and other materials, to maintain stiffness and increase wear resistance. These so-called hybrid gears—which feature steel in the teeth and hub and can reduce weight by as much as 40% compared to standard all-steel gears—are the subject of several papers [6,7,8]. Handschuh et al. [6] conducted an experimental study in which hybrid gears consisting of steel teeth and a steel inner hub were connected by a composite web. A strong dependence of the operating speed on the vibration level was found, indicating the importance of the targeted use of composite materials to reduce vibrations and weight. Similarly, Catera et al. [7] investigated a steel-composite hybrid drive, where the steel teeth and internal hub are connected by a composite web. In their studies, a multiscale modelling approach was employed to accurately estimate the natural frequencies of the hybrid transmission, taking into account the unique properties of composite materials. In their follow-up study, Catera et al. [8] conducted a numerical investigation that implemented a thin steel layer to establish a connection between the steel teeth and the inner hub, successfully addressing the issue of centreing problems. Additionally, a composite web was incorporated into a steel gear body. A comparative analysis was conducted between hybrid steel gears with identical macro geometry and mass. The static transmission errors of the hybrid gear and the lightweight steel gear were calculated using FEM. The results indicated a considerable decrease in peak-to-peak static transmission error, leading to an enhanced performance of the hybrid transmission in terms of noise, vibration, and harshness (NVH). It is worth noting that the composite material used in these studies served solely as a connection between the steel teeth and the inner hub, aimed at reducing weight and improving NVH performance.

For low load capacity, the injection moulding process efficiently provides lightweight polymer gears that do not require lubrication. To increase load capacity and wear resistance, short reinforcing fibres are embedded in a polymer matrix. Short fibre (SF) composites are materials that contain fibres that are typically less than 1 mm long. Blais and Toubal [9] investigated HDPE gears reinforced with short natural fibresand subjected to high-cycle bending fatigue. Their study shows that injection moulding of HDPE/birch fiber is a promising process to manufacture eco-friendly machine parts at lower costs. Mohsenzadeh et al. [10] investigated the load-bearing characteristics of polyoxymethylene (POM) with carbon black (CB) CaCO_3_ ternary nanocomposite gears using a temperature-based step loading approach with a 30% increase of mechanical properties. Furthermore, the SEM micrograph of worn surfaces revealed a much smoother worn surface of composite gears, which indicates beneficial synergistic strengthening effects of CB and nano-CaCO_3_ composite gears. Kurokawa et al. [11] tested poly-ether-ether ketone (PEEK) gears reinforced with 15% CF under different test conditions, i.e., dry running and lubricated running, and in combination with a driven steel gear. For comparison, the PAI-CF30 and PPS -GF30 gears were also injection-moulded and tested under non-lubricated conditions, with the driving and driven gears made of the same material. The PEEK + 15% CF gears showed the best performance compared to the other two high-performance plastics. In their subsequent study, Kurokawa et al. [12] tested injection-moulded polymer gears to determine their performance. The gears were made of various types of polyamides (Pas) reinforced with short carbon fibres, including PA12, PA6, and PA46 with a 15% addition of carbon fibres. The tests were conducted under grease-lubricated conditions, with the driving and driven gears made of the same composite material. The results indicated that the PA46 gears experienced wear in a relatively short period of time while the carbon fibre reinforced PA12 and PA6 gears failed due to fatigue. The gears made of short carbon fibre reinforced PA12 exhibited the best performance, with results comparable to PEEK-CF.

**Figure 1:**
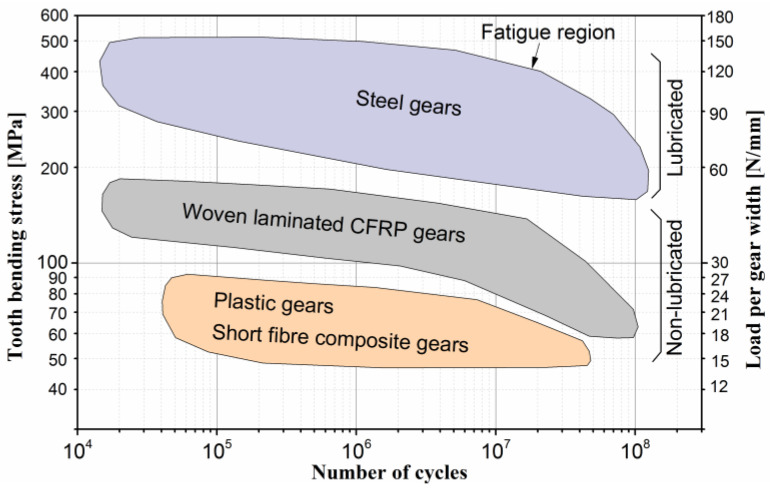
Fatigue region for different classes of materials; woven laminated CFRP gears fill the large gap between steel and plastic gears and composite gears with short reinforcing fibres.

Incorporating reinforcing particles has been demonstrated to enhance wear resistance significantly. Mao et al. [13] found a significant reduction in specific wear rate and a 50% increase in load-bearing capacity by introducing 28% glass-reinforcing particles into POM (polyoxymethylene). Normally, the static tensile strength and modulus of elasticity can be increased by adding short reinforcing fibres. However, for dynamically loaded components, polymer gears without short fibre reinforcements can perform better than the composite gears reinforced with short fibres [14]. While the fibres increase the modulus of elasticity and the quasi-static strength of the composite, the interfaces between the hard fibres and the soft matrix can become areas of microscopic stress concentration and, thus, act as crack initiators during fatigue loading [15,16]. On the other hand, there are many known benefits of continuous fibre reinforced composites, such as balance of properties, higher stiffness and strength, higher toughness, creep and fatigue resistance, dimensional stability, etc. Load, applied to a fibre reinforced composite material, is transferred from matrix to fibres by shear stresses along the fibre-matrix interface and reaches a maximum when fibres are aligned in the load direction; transfer efficiency increases with the increase of fibre length-to-diameter ratio [17]. The composite laminate is an assembly of layers of fibrous composite materials which can be joined to provide the required engineering properties, including in-plane stiffness, bending stiffness strength, and high modulus. Typically, individual layers are orthotropic or transversely isotropic with the laminate exhibiting orthotropic or quasi-isotropic properties. The failure types of autoclave-cured, woven laminate composite gears and their fatigue properties were investigated by Zorko et al. [18,19]. The two failure modes were presented: two–three body abrasive wear, and fatigue. A 6-times longer lifespan at the same fatigue load was observed for the woven CFRP gears compared to the high-performance PEEK gears. In comparison with other gears made of polymers or short fibre reinforced polymer composites, the load bearing capacity was even higher. The fatigue regions between different classes of materials are shown in Figure 1. The autoclave-cured, woven CFRP gears show excellent potential to close the large performance gap between the polymer and steel gears. Their ability to run without lubrication, excellent strength-to-weight ratio, and good damping characteristics make them promising candidates for aerospace applications.

Gear wear and fatigue performance are highly dependent on the tribological behaviour of the gear pair at the meshing contact interface. Bijwe and Sharma [20] conducted a study on the potential use of CFRP in tribological applications. They investigated how varying the carbon fibre content ratio in CFRP with a polyetherimide (PEI) thermoplastic matrix affected its mechanical and tribological properties. The study revealed that an optimal combination of mechanical and tribological properties was achieved with a CFRP containing 65% fibre content. Ramesh and Suresha [21] analysed the frictional and wear characteristics of epoxy-impregnated carbon fibre reinforced polymer (CFRP). Their findings indicate that, when subjected to abrasive wear, carbon fabrics demonstrate superior performance compared to glass fibres and other types of composite. The researchers observed a further reduction in surface wear when the CFRP was combined with aluminium oxide (Al_2_O_3_) and molybdenum disulphide (MoS_2_). The findings suggest that, by optimising the carbon fibre content ratio and incorporating wear-resistant particles, such as Al_2_O_3_ and MoS_2_, CFRP has the potential to enhance its tribological behaviour and minimise surface wear. Moreover, Černe et al. [22] studied the thermomechanical response of the laminated composite gears, where they proposed a new iterative method for implicit evaluation of coefficient of friction (COF) between the woven CFRP gear and its steel counterpart.

Ekoi et al. [23] analysed the static and fatigue behaviours of additive manufactured continuous carbon fibre reinforced polymer (CFRP) composites, and compared the woven and non-woven structures. Their investigation also explored the failure mechanisms associated with fatigue in these composites. The fatigue test results revealed that woven carbon composites outperformed their unidirectional (non-woven) counterparts when subjected to 70% of their ultimate strength. In the case of non-woven composites, failure was observed in multiple regions with long splitting of the composite and fibre breakage. On the other hand, for woven composites, failure mostly occurred due to fibre pull-out at the interface between the unit cells, which is attributed to stress concentration.

This study presents a comparative analysis of the fatigue and wear properties of gears made from carbon fibre reinforced polymer (CFRP) fabricated through different methods, including autoclave-cured, prepreg, and vacuum-infused techniques. Additionally, the present study aims to investigate how the durability of gears is affected by different types of fibres, resin, and areal weights of fabrics.

An autoclave is a type of pressure vessel that uses heat and pressure to cure materials. The process typically takes place at pressures between 4 and 8 bar and temperatures between 100 and 250 °C. The parts are typically made from a material called prepreg, which is a fabric that has already been pre-impregnated with a catalysed resin. Prepreg resins are typically partially cured or B-staged, meaning that they have already undergone a partial curing process before they are ready to use. The prepreg is sealed in air-tight plastic bags and stored at −18 °C before use. Before lamination of the prepreg, it is brought to room temperature and cut into different shapes to fit the desired mould. The material is then covered with a peel ply, perforated foil, and a felt or breather (absorbent ply), and sealed in a vacuum bag. When composite materials are subjected to pressure in an autoclave, any air bubbles present within the material are forced out of the laminate. The process of removing air bubbles is called “outgassing”. The air bubbles can escape through any small openings in the laminate, such as the edges of the mould or through perforations in the vacuum bagging materials. The vacuum pump also creates a low-pressure environment in the autoclave chamber which helps to evacuate the remaining air bubbles in the laminate. In the end, the final composite material is free of bubbles, which improves its mechanical properties such as strength, stiffness, and toughness. The autoclave manufacturing method is used to create high-quality structural parts for the aerospace industry [24,25].

Vacuum infusion is a process used to produce composite parts by impregnating the fibres with a resin. Unlike prepreg resins, vacuum infusion resins are usually fully uncured or A-staged, and undergo complete curing during the vacuum infusion process. The process involves laying the fibres inside a form and then creating a vacuum to pull the resin through the fibres and into the shape. Once the form is filled with resin, it is then cured under heat and pressure to create a finished composite part. The main advantage of vacuum infusion over traditional layering techniques is that it allows for more precise control of the resin-to-fibre ratio and can result in more consistent and higher-quality parts.

The main difference between the two processes is that vacuum infusion impregnates dry fibres and employs vacuum to pull the resin into the mould, while autoclave curing requires pre-impregnated fibres, which have to be heated and pressurised to cure the part. Although the vacuum infusion has many benefits, to completely remove the air from the dry preform, the available resin choice is limited to non-toughened resin systems. This research aims to investigate the durability of carbon fibre reinforced polymer (CFRP) gears made from both toughened and non-toughened resin systems. Toughened epoxy systems are often used by manufacturers in the production of prepreg materials to improve the fracture toughness of the final product. However, the specific techniques used for the toughening of prepreg resins systems are typically not disclosed, although they often involve incorporating reactive oligomers such as carboxyl-terminated butadiene–acrylonitrile (CTBN) rubber or pre-formed elastomer particles [26,27].

Overall, this paper presents unique insights that have not been widely covered in the literature, highlighting the potential for woven as well as the continuous 3D printed CFRP composites to revolutionise the field of powertrain systems. The recognition and adoption of the findings from this research have the potential to generate positive environmental effects, such as increasing the durability of powertrain components and reducing wasted plastic. By incorporating CFRP composites into powertrain systems, these components can have longer lifetimes, low maintenance costs, and require less frequent replacements, ultimately reducing waste and the associated environmental impact.

With a suitable processing technique, CFRP gears can exhibit high mechanical strength, good thermal stability, high thermal conductivity, and favourable tribological properties. This makes woven laminated CFRP gears an ideal candidate for use in complex technical systems such as gearboxes and other power transmission components. Opportunities exist to improve the thermomechanical and tribological properties of composite laminated gears and achieve superior performance through further research and development.

## 2. Materials and Methods

### 2.1. Gears’ Production

For this investigation, we utilised different standard modulus PAN-based carbon fibre (Toray Industries Inc., Tokyo, Japan) The fibre type, tex, areal density, ply thicknesses, and mechanical properties are listed in Table 1. The fibre type T300 was used as a baseline carbon fibre, which is commonly employed in aerospace applications with a tensile strength of 3530 MPa and an elongation at break of 1.5%. Furthermore, a comparison with a T700S fabric was carried out, which is a higher-quality fibre known for its strength in high-stress applications, with a tensile strength of 4900 MPa and an elongation at break of 2.1%. In all cases, plain weave fabrics with equal properties in warp and weft directions were used.

Table 2 lists the epoxy resins used in the pre-impregnated (prepreg) fibres and in the vacuum-infused plate. Prepreg fabrics were impregnated by the selected manufacturers, whereas the vacuum infusion was performed in the authors’ laboratory. The E445 epoxy-prepreg resin system (Composite Materials s.l.r., CIT, Legnano, Italy) is a structural epoxy, specifically designed to achieve an excellent surface finish of the cured part. It can be polymerised through autoclave, vacuum bag, and hot mould processes. The DT120 epoxy-prepreg resin DeltaPreg (Delta Tech S.p.A., Toray Group, Lucca, Italy) is a toughened epoxy from designed for autoclave processing, which is intended for applications that require higher fracture toughness. Last, the LG 900 UV, the standard 2-component (liquid) epoxy (GRM Systems s.r.o., Olomouc, Czech Republic), is used for wet-layup, vacuum infusion (VI or VART), and resin infusion (RTM) processes. It is cured at room or at slightly elevated temperature, followed by post-curing at a temperature range between 80 and 110 °C.

The gears were produced from laminated panels with dimensions of 180 mm × 180 mm. With a purpose to compare different production processes, fabrics, and matrices, three different plates were fabricated. The summary of the plate manufacturing and stacking sequences is given in Table 3.

**Figure 2:**
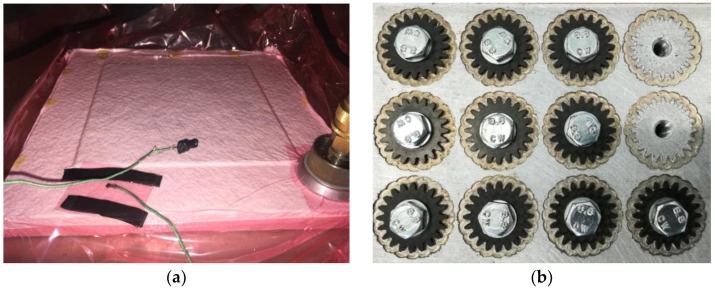
Production technology of CFRP gears (**a**) laminated plate in vacuum before applying a pressure; (**b**) after clamping and milling operation to produce CFRP gears from laminated plate.

The first panel was made using prepreg layup and vacuum bagging, followed by autoclave consolidation and curing. It comprises 8 layers of 200 g/m^2^, 3K plain weave carbon fibre (CC202, 3K, 200 g/m^2^, Torayca T300, Toray Industries, Inc., Tokyo, Japan), pre-impregnated with resin ET445 (Composite Materials, s.r.l., CIT) with a quasi-isotropic stacking sequence [(45/0)_2_]_S_ which is a 8-ply laminate [45/0/45/0¦0/−45/0/−45]. In the used designation, each number represents the longitudinal warp orientation of the ply, e.g., 45 represents a woven ply oriented with warp (+45°) and weft (−45°) directions. The laminate was covered with a peel ply and a 3 mm thick non-woven Breatex absorber to ensure uniform pressure distribution across the entire surface. The produced panel, placed in the autoclave with attached thermocouple to regulate the curing temperature, is shown in Figure 2a. The pressure of 400 kPa was applied in an autoclave to consolidate the layers and ensure proper adhesion. The heating rate was set at 3 °C/min and the panel was cured at a temperature of 130 °C for 100 min. The mechanical characteristics of the used material have been thoroughly documented in previous work by the authors [24].

The second panel was also made using the autoclave process. It comprises 18 layers of 1K plain weave carbon fibre (6609P, 93 g/m^2^, T700S, Toray Industries, Inc., Tokyo, Japan) pre-impregnated with toughened resin DT120 (Delta-Tech, Toray Group, Lucca, Italy). The production of thin-layered fabric is slow and, therefore, more expensive than normal 3K fabrics. It is used for obtaining the strongest and densest composites. To achieve the approximate thickness of the previous sheet, the number of layers was increased to 18. This prepreg material is used for special applications where high strength in different directions is required. To achieve strength in multiple directions, the fibre orientation changed by 22.5° increments (90°/4 = 22.5°). Therefore, the fibres were placed in 0°, 22.5°, 45°, 67.5°, and 90° direction. This method maximised the proportion of 0° layers in each tooth. The curing procedure of the second panel was identical to that of the first panel.

**Figure 3:**
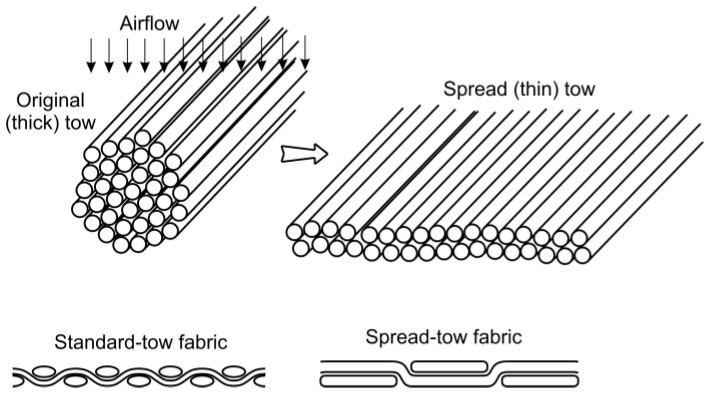
Difference between standard and spread-tow fabric after spread technique [28].

The third panel was made using vacuum infusion technology and subsequently cured in the autoclave. It comprises 18 layers of 1K plain weave carbon fibre, designated as 469 spread-tow fabrics, 67 tex (C. Cramer GmbH & Co. KG, Heek, Germany), vacuum-infused with laminating resin LG 900 UV with HG120 hardener (GRM Systems, Olomouc, Czech Republic). The stacking sequence is the same as that used in the second plate. There are several known technologies to make a spread tow. Spread-tow carbon fibre fabric is a dry carbon fibre reinforcement made using a spreading technique to flatten the tows of the carbon fibre, which can be accomplished using a stream of air and the pre-tension [28]. The result is a fabric that is smoother and flatter than conventional woven fabric, with less crimp that occurs at the intersection of warp and weft: Figure 3. Spread-tow fabrics are often used to create thin-ply laminates with superior mechanical properties compared to standard-ply laminates [29]. In thin-ply laminates, more layers per unit thickness allows for higher variation in ply angle orientations and, therefore, could potentially improve load-bearing properties. The epoxy LG 900 UV laminating system includes UV inhibitors, but, unlike the epoxy used in panel 2, it is not toughened. After vacuum infusion, the panel was placed into the autoclave, where the pressure of 400 kPa was applied. The cure cycle for this resin was 15 h at 40 °C for initial polymerisation, then a ramp of 0.5 °C/min to 80 °C for 3 h, and then a ramp of 0.25 °C/min to 110 °C for 5 h.

The CFRP gears were milled from panels using a Sodick CNC machine. Central holes (6 mm) were milled first; then, the CFRP panels were bolted to an aluminium base plate for high-quality gear edge cuts as shown in Figure 2b.

The steel gears were milled from tempered steel EN 42CrMo4 (W.No. 1.7225, AISI/SAE 4142) which has a chemical composition of 0.41% of C, 0.2% of Si, 0.75% of Mn, 01.05% of Cr and 0.23% of Mo, and then plasma-nitrided. They were treated with a superfinish to smooth surfaces and remove sharp edges. The flank profiles were measured with a MarSurf XC20 conturograph and the surface roughness was measured before the test with a Tesa Rugosurf 90G gauge. The surface roughness of the superfinished steel gears was Ra = 0.689 μm and the surface roughness of the CFRP gears was Ra = 0.417 μm.

**Figure 4:**
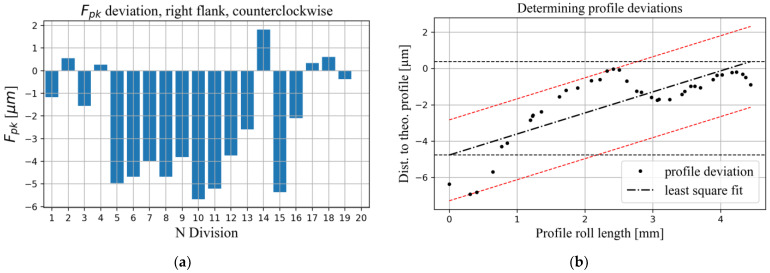

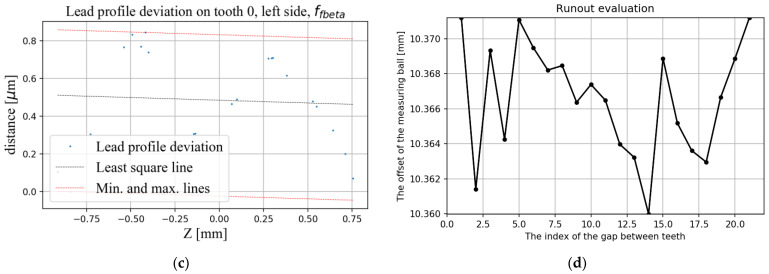
Exemplary results depicting the evaluation of gear quality parameters according to ISO 1328 based on 3D-scanner measurements. (**a**) cumulative pitch deviation; (**b**) profile deviations; (**c**) lead profile deviations; (**d**) gear runout.

The surface hardness of the steel gears was measured using a standard method and an average value of 870 HV_0.2_ was obtained. A cylindrical spur gear geometry with parameters defined in Table 4 was used in the manufacturing process. After manufacturing, the geometry of the carbon fibre reinforced plastic (CFRP) gears was measured with an ATOS Compact SCAN 5 M scanner with a nominal accuracy of ±2 μm. The geometric quality of the gears was evaluated in accordance with ISO 1328 using a self-developed software for quality control of gears. The results indicated that the pitch quality was level 7, the profile quality was level 9, the pitch lead profile quality was level 9, and the runout quality was level 6. Representative results from the carried-out gear inspection, following the methodology described in Ref. [30], are presented in Figure 4.

### 2.2. Thermomechanical and Frictional Characteristics of the ET445 CIT Composite

**Figure 5:**
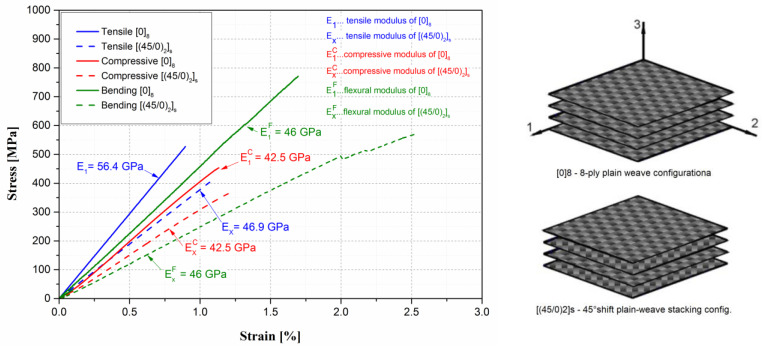
Quasi-static mechanical characterisation results for the produced ET 445 CIT autoclave prepreg.

The T300-ET445-200tex plain weave prepreg was the benchmark composite; therefore. it has been thoroughly characterised in terms of its mechanical and thermal properties. Initially, the mechanical properties in quasi-static load conditions have been evaluated, using the procedures thoroughly described in Ref. [24]. The results are shown in Figure 5. As is common for this type of composite, highly variable mechanical properties have been identified, depending on the fibre layup configuration and load direction. On the other hand, a fairly linear stress–strain relation has been identified for all tested load conditions and composite configurations. The measured mechanical properties of the [(45/0)_2_]_s_ layup configuration, which is the one used for the carried-out gear production and testing, are noted in a more complete form in Table 5.

The thermal characteristics of the [(45/0)_2_]_s_ ET445 CIT prepreg have additionally been analysed using a TPS 1500 Hot Disk Thermal Constants Analyser. In the in-plane fabric direction, a thermal conductivity of 2.416 W/(mK) was measured, along with 2138 J/(kgK) specific heat capacity. In the traverse, out-of-plane direction, these properties changed to 1.446 W/(mK) and 4106 J/(kgK), respectively, pointing to an orthotropic thermal behaviour as well.

A key property that can influence the performance and efficiency of a gear pair is the coefficient of friction (COF). The tribological properties of the [(45/0)_2_]_s_ ET445 CIT prepreg CFRP have been assessed using a reciprocating cylinder-on-flat tribological test setup, with a 42CrMo4 cylinder sliding on the flat side surface of the CFRP. Using this type of test setup, an average COF of 0.34 has been identified, which is comparably slightly higher than typical values observed for steel thermoplastic pairs such as steel-POM or steel-PA6. The measured COF value has also been confirmed by applying the implicit gear-pair COF identification model presented in the previous study [22].

### 2.3. Gear Testing Methodology

**Figure 6:**
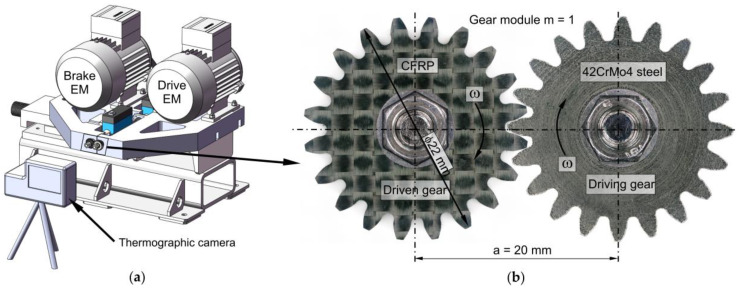
Experimental set-up for reference gear pair testing. (**a**) testing rig; (**b**) CFRP gear in mesh with steel pinion.

Gear tests have been carried out to measure the developed gears’ service life as a function of load and identify and characterise the predominant failure modes at the considered loads. The used test rig consists of two electric motors, one of which serves as a drive motor and the other as a brake electric motor. The test stand allows the torque to be continuously adjusted via a drive motor and a brake motor which are controlled via variable frequency drives. The drive motor and the brake motor are four-pole asynchronous electric motors (Siemens AG, Munich, Germany) with a nominal torque of 2.5 Nm. Power is transmitted from the drive electric motor to the drive shaft of the gearbox via a toothed belt that dampens the vibrations of the electric motor. The testing rig has Futek TRS600 torque sensors and speed sensors mounted on the drive and driven shafts, enabling continuous torque measurement and closed-loop control. The test rigs were positioned in a thermal chamber where the ambient temperature and humidity were controlled. More details about the testing unit can be found in previous studies [31,32]. The CFRP gear geometry and dimensions in a mesh with driving 42CrMo4 gear are presented in Figure 6.

### 2.4. Scanning Electron Microscopy (SEM)

The surfaces of the worn gears were examined using a Thermo Fisher Scientific Quattro S scanning electron microscope (SEM) equipped with an Everhart–Thornley detector (ETD) and a circular backscatter detector (CBS). SEM images of the carbon fibre reinforced plastic (CFRP) gear surfaces were taken before and after the tests. The elemental composition of the worn CFRP surface was investigated using energy-dispersive X-ray spectroscopy (EDS), and elemental mapping was also carried out to investigate the distribution of elements on the surface.

### 2.5. Temperature Measurements

**Figure 7:**
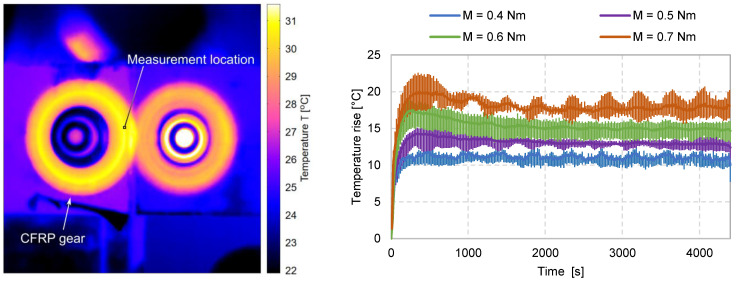
Temperature rise above ambient temperature (22 ± 2 °C), measured below the tooth root using a thermographic camera during the carried-out gear tests.

During gear running, a certain temperature rise on the meshing contact interface (flash temperature) and the gear body (bulk temperature) is always present due to frictional and hysteresis losses at the contact and in the material structures. An overly elevated temperature during gear running is generally a strong indicator of unfavourable frictional characteristics of the used gear pair, or unsuitable thermal conductivity or heat transfer via the surrounding medium. The bulk temperature of the tested CFRP gear was measured using a FLIR T 420 thermal imaging camera. The measurement was taken in an area of 3 × 3 pixels (ROI) in the root area of the gear. To minimise infrared reflection (IR) from the background surfaces, the surfaces behind the gears were coated with a layer of Acktar Metal Velvet TM. The camera was calibrated by initially setting the emissivity to ε = 0.95 and subsequently recalibrating the measurements based on the evaluated mean emissivity. The emissivity of the analysed CFRP sample in the temperature range between 30 and 72 °C was measured to be, on average, *ε*_t_ = 0.935; however, a temperature-dependent behaviour was observed with the emissivity values approximately following the function *ε*_t_(*T*) = −0.0006 *× T* + 1.1268 (with temperature in K). The measurement results are shown in Figure 7. The temperature is plotted as the increase above the ambient temperature, which was 22 ± 2 °C during all carried-out tests. Evidently, for the selected gear pair configuration and loads, the heat losses are not overly high and do not result in temperatures that could lead to any thermally induced damage mechanisms.

### 2.6. Wear Measurement

For the wear monitoring tests, the CFRP gears were tested over a period of five intervals, each lasting 24 h (for a total of 120 h). At each interval, the wear was measured using image analysis of the worn surfaces. The images for the wear analysis were taken using a Keyence VHX-6000 3D digital microscope at 50× magnification. The images were then imported into AutoCAD software for analysis. The original image of the unworn gears was compared with the image of the worn gears to determine the amount of wear. The wear measurement was taken on every other tooth, for a total of 10 teeth of each gear.

### 2.7. Numerical Analyses

**Figure 8:**
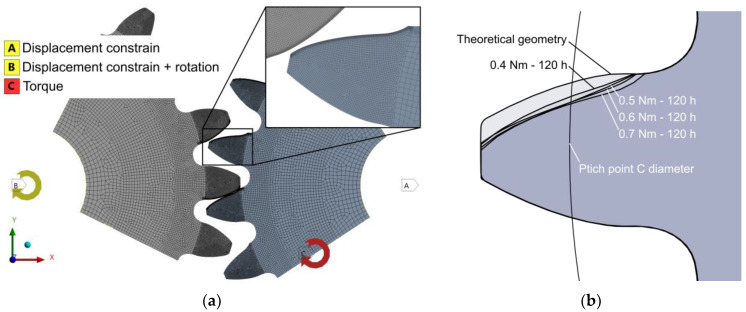
Constructed finite element model with depicted FEM mesh and applied boundary conditions (BCs) and loads. (**a**) FEM model geometry, mesh, BCs and loads; (**b**) considered gear profile geometries.

To analyse the impact of wear on the gear-meshing kinematics and mechanical behaviour of the material pair, numerical finite element analyses were performed using both the theoretical geometry and the worn gear geometries, obtained from microscopy measurements of the tested samples. Samples that had run at loads between 0.4 Nm and 0.7 Nm for 120 h were used. Figure 8 shows the developed finite element model and the gear geometries used. The model, built in ANSYS Workbench 2022 R1, employs 2D plane-stress and quasi-static mechanical behaviour assumptions, and enables a time-dependent contact analysis of the entire gear-meshing process, using isotropic/orthotropic linear elastic models for steel and CFRP, respectively (CFRP orthotropic parameters based on data given in Table 3), along with geometric and contact nonlinear mechanical models and a specified COF of 0.34 [22].

## 3. Results

### 3.1. Wear Characteristics of CFRP Gears

**Figure 9:**
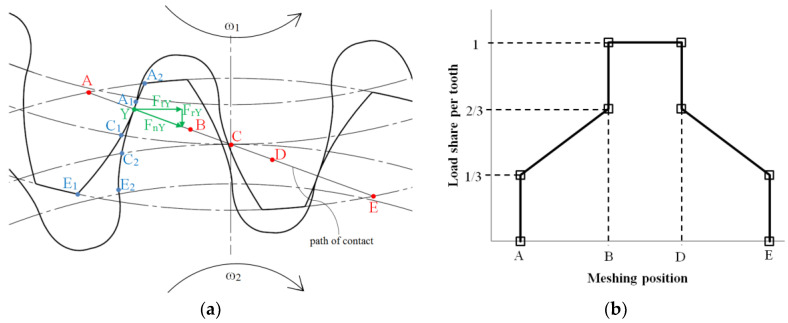
Meshing: (**a**) theoretical meshing of the tested gear pair geometry; (**b**) theoretical load sharing for the tested gear geometry.

The primary cause of gear wear is the abrasion resulting from the friction generated by the meshing of the gears. The meshing cycle for a selected pair of teeth goes from points A to E (Figure 9). Point A is the initial meshing point; this is point A1 on the drive gear’s flank and A2 on the driven gear’s flank. Point B is the lowest point of single tooth contact (LPSTC), and point D is the highest point of single tooth contact (HPSTC) for the driving gear. For the driven gear, the situation is vice versa; point D is the LPSTC, and point B is the HPSTC. When gears mesh from points B to D, the entire load is transmitted via a single tooth pair. When meshing in the regions from A to B and D to E, the load is transmitted over two pairs of teeth. This phenomenon is known as load sharing (Figure 6) and is extremely important for understanding the load on a single tooth. Finally, point E is the endpoint of contact. Spur gears in operation are subjected to torque, which results in a normal force *F*_nY_ acting in an arbitrary meshing point *Y* between the two teeth in contact. The normal force *F*_nY_ can be decomposed into radial *F*_rY_ and tangential force *F*_tY_. In involute gear pairs, the normal force acts along the path of contact. The magnitude of torque applied was selected on the fact that polymer gears have maximum load per gear width of 20 N per 1 mm.

In our previous studies [15,16], the experimental region to test gears was determined for CFRP ET445 CIT with plain weave carbon fabrics, with an areal mass of 200 g/m². The upper limit of the torque applied was found to be 0.8 Nm, beyond which failure occurred almost immediately. For comparison, the maximum transmittable torque for steel gears of the same size ranges from 1.7 Nm for through-hardened and tempered C45 steel to 3.5 Nm for case-hardened 18CrNiMo7-6 steel, with a service life of 10^7^ load cycles.

**Figure 10:**
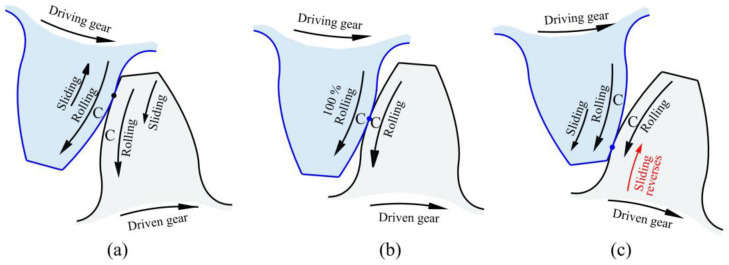
Meshing (**a**) sliding and rolling; (**b**) only rolling; (**c**) rolling and reversed sliding.

Moreover, the wear in spur gears is caused by the combination of sliding and rolling contact. The sliding is most severe at the tip and just above the root of the CFRP gear tooth, gradually decreasing until only rolling occurs at pitch point C. Pitch point C marks the threshold where the direction of sliding changes as the meshing moves past this point, as illustrated in Figure 10.

**Figure 11:**
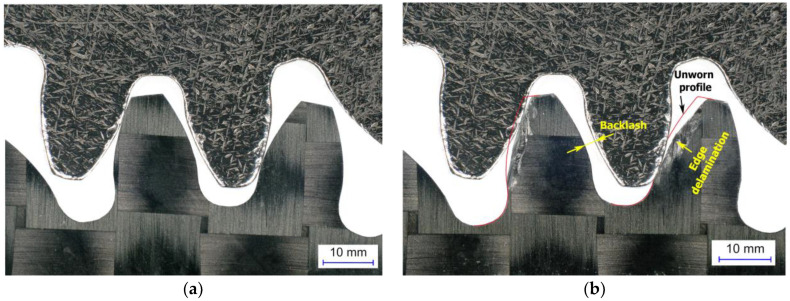
Meshing of the testing gears, steel driving gear with CFP-driven gear (**a**) new CFRP gear in meshing; (**b**) worn CFRP gear after 120 h of operation (1 × 10^7^ cycles) at torque of 0.4 Nm.

**Figure 12:**
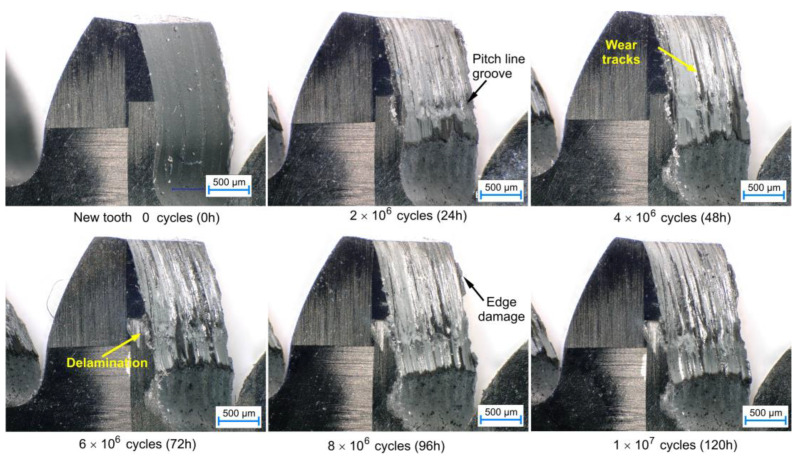
Wear degradation of gears of sample CFRP gear, tested at torque of 0.4 Nm.

As shown in Figure 11, wear results in an increase in gear backlash, i.e., the clearance between the passive tooth flanks of the two meshing gears. It can also significantly impact the meshing kinematics and the resulting strain-stress state in the gear structure. Furthermore, the surface ply-delamination is visible on the CFRP edge of the working flank. Figure 12 displays a series of images taken after 24 h intervals during testing, clearly demonstrating the gradual degradation of the material. The images reveal typical wear patterns on the tooth flanks, including the groove at the pitch diameter, increased surface roughness, edge delaminations, and extruded/intruded ply interfaces.

**Figure 13:**
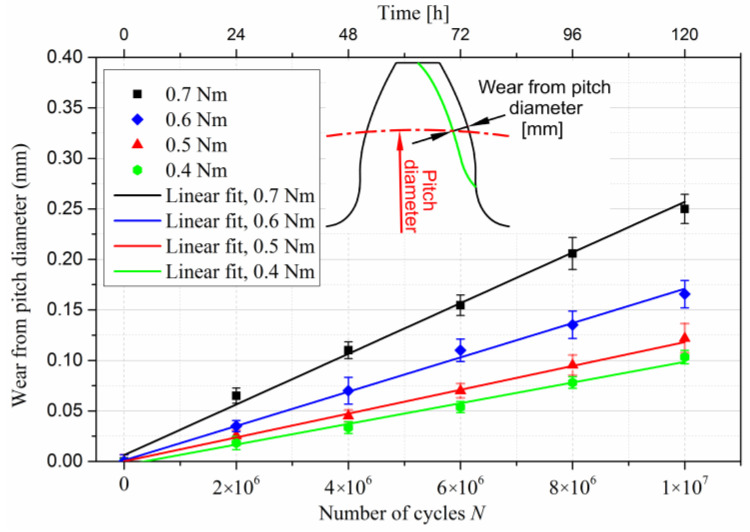
Wear from pitch diameter against the number of cycles.

Figure 13 shows the relationship between wear from the pitch line and the number of cycles for four different loading conditions. Each datum point is the average wear volume on 20 gear teeth, with error bars representing 95% confidence intervals. The gears were tested to 10^7^ cycles, the expected lifespan for common polymer and composite gear applications. Wear at the pitch diameter is the normal distance from the worn to the unworn tooth profile. There is a clear correlation between wear rate, load, and testing time. Wear from pitch diameter increased linearly with increasing cycles and torque.

### 3.2. Numerical Analysis

**Figure 14:**
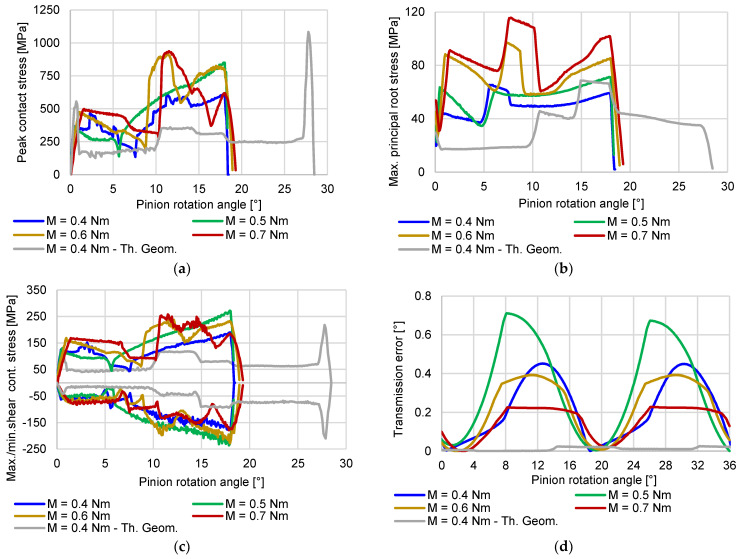
Main FEM analysis results obtained for the theoretical gearing geometry and worn sample geometries at various loads after 120 h of testing, recreated based on microscopy measurements. (**a**) Peak contact pressure; (**b**) Peak root stress on active flank side; (**c**) Peak shear stress below contact; (**d**) Transmission error.

The results of the numerical analysis are shown in Figure 14.

**Figure 15:**
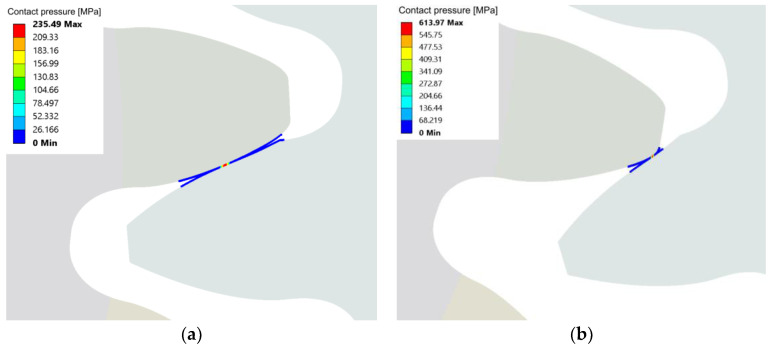
Contact pressure evaluated for the 0.4 Nm load worn geometry (after 120 h running) at (**a**) approximately the pitch diameter contact C, and (**b**) at the location of peak pressure during the simulated meshing cycle.

Evidently, wear has a significant impact on the stresses during gear meshing. Comparing the peak contact stress (Figure 14a) between theoretical and worn geometry at 0.4 Nm, we can see that the peak pressure noticeably increased, especially in the second phase of meshing, i.e., after the pitch point contact is reached. A graphical presentation of the pitch point contact pressure and overall peak-evaluated pressure for the 0.4 Nm worn gear geometry load case is additionally shown in Figure 15. The described pattern is exhibited, and is even more pronounced at higher loads, where the wear level was higher. A comparison of the root stress results between the theoretical and worn geometries (Figure 14b) reveals a slight decrease in stress on the worn geometry compared to the theoretical geometry, with the stress pattern altered and exhibiting stress spikes at the start and end of the meshing cycle.

**Figure 16:**
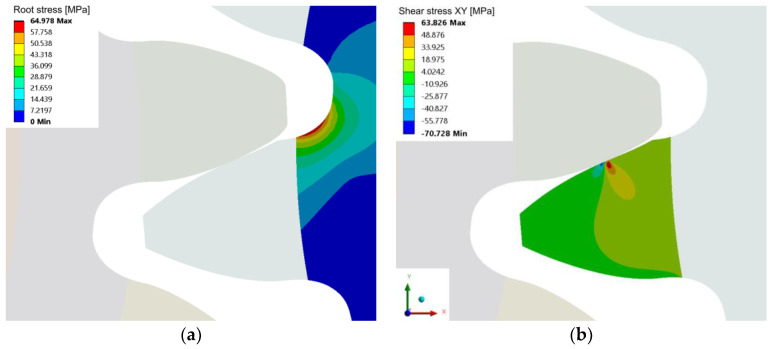
FEM results at 0.4 Nm torque, (**a**) the root stress on the active flank side evaluated as max. principal stress, and (**b**) the shear stress at and below the contact area. Both results are plotted ap-proximately at the pitch diameter point C.

Similar to the contact stress, the peak shear stress below the contact area (Figure 14c) shows an increased level in the second half of the meshing cycle, where the magnitudes exceed the theoretical geometry values. For a clearer overview, the root and shear stresses at approximately the pitch point contact C are depicted in Figure 16a,b respectively.

Figure 14d also shows that the transmission error increases substantially compared to the theoretical profile geometry, even for the lowest wear level, as measured for the 0.4 Nm load case. The error, however, does not increase linearly with the load and wear levels, pointing to the necessity for a more in-depth investigation of this correlation. The presented results also point to the fact that wear noticeably decreases the overall length of the meshing cycle. For the 0.4 Nm load, the duration of the meshing cycle is, e.g., reduced by 29.3% compared to the theoretical geometry.

**Figure 17:**
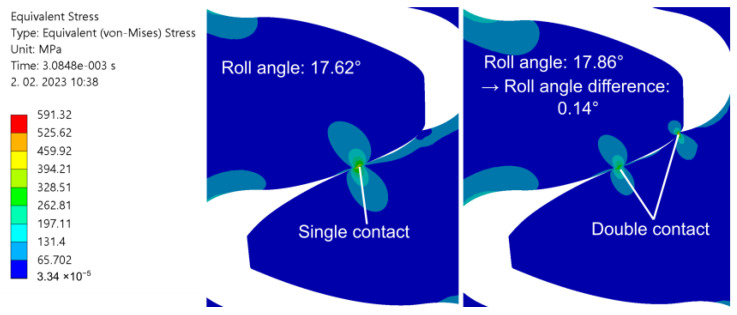
Transition from pitch area contact to double contact, as observed on all worn CFRP gear sample analyses. The depicted case was evaluated for M = 0.7 Nm/120 h sample geometry.

The numerical simulations with the worn gear geometry also showed that the path of contact diverges substantially from the theoretically predicted linear. A transition of the contact location was observed from the area near the pitch diameter to the bottom part of the CFRP gear profile (near the base diameter) in the second half of the meshing phase. This caused a sudden change in the contact path and a significant increase in the contact pressure on the bottom part of the CFRP tooth flank, which may further speed up the wear process. During the described transition of the contact location, a short period of double contact near the pitch and base diameters was observed, which is shown in Figure 17. This behaviour was observed in all four analysed worn gear geometries, with loads ranging from 0.4 Nm to 0.7 Nm. The wear level can result in detrimental impacts on the contact stress state, negatively affecting the performance of the gears. This can also lead to an increase in noise and vibrations.

### 3.3. Fatigue Properties of CFRP

One objective of the study was to improve the fatigue properties of CFRP gears beyond the achievements of our previous research [19]. To achieve this, higher quality fibres, using thinner plies, additional fibre orientations, and a toughened epoxy resin formulation were used, as described in Section 2.1. For instance, replacing T300 with T700S fibres can improve strength and potentially reduce wear rates. By utilising plies that are more than half as thin (0.11 mm instead of 0.26 mm), the load distribution between plies can be improved, and the stress at the interface regions can be reduced. Increasing the number of plies for the same thickness of gears allows for a wider range of fibre orientations, which maximises the content of parallel fibres in each tooth. In addition to 0°, 45°, and 90° plies, we used inter-plies of 22.5° and 67.5° to decrease the angle increment between plies. Additionally, a toughened epoxy formulation can decrease wear rates, enhance fracture toughness at the interface regions, and reduce the crack initiation at the fibre–matrix interfaces. 

**Figure 18:**
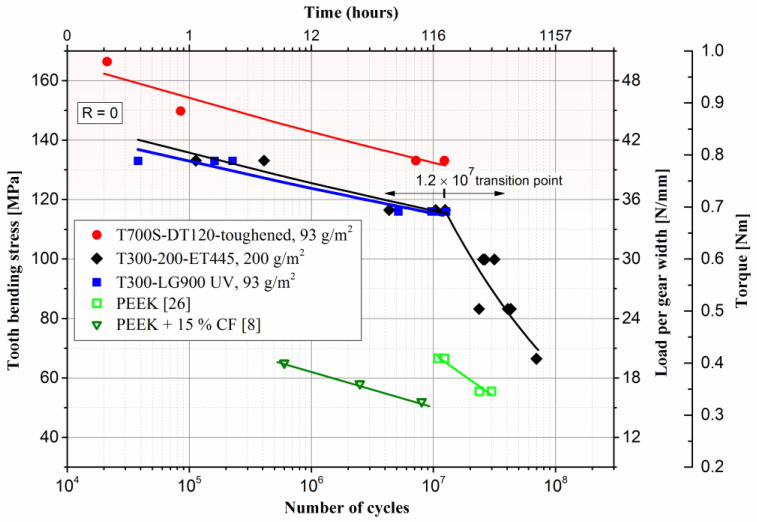
Maximum tooth bending stress against the number of cycles for the CFRP gears and in comparison with PEEK gears.

The fatigue properties of CFRPs made from the autoclave-processed and vacuum-infused panel are presented in Figure 18. The graph features multiple scales on the *y*-axis to facilitate a thorough evaluation of the material’s fatigue characteristics. The primary *y*-axis displays the tooth bending stress, which is a commonly used metric in gear fatigue testing. The secondary *y*-axis shows the load per gear width, which provides information on the loading conditions of the gears during testing. The third supplementary *y*-axis displays the corresponding torque.

The VDI equation 2736 [33] is a widely accepted and recognised standard for calculating tooth bending stress in gear fatigue testing. It utilises a mathematical model that considers the rigid gear geometry and the isotropic linear elastic behaviour of the material. The equation accounts for the geometric properties of the gear, such as tooth profile and backlash, as well as the material properties, such as elastic modulus and Poisson’s ratio, in order to predict the tooth bending stress under a given loading condition. Tooth bending stress is calculated as:

(1) $$\sigma_{F}=K_{F}\times Y_{\mathrm{Fa}}\times Y_{\mathrm{Sa}}\times Y_{\varepsilon }\times Y_{\beta }\times \frac{F_{t}}{b\times m_{n}}$$

where the following factor values were used for the tested gear geometry: *K*_F_ = 1.0; *Y*_Fa_ = 3.01, *Y*_Sa_ = 1.51; and *Y*_ε_ = 0.732; *Y_β_* = 1. To evaluate the performance of the gear and to compare results with data from the literature, the load per gear face width, also known as *F*_w_, is plotted on the second *y*-axis. Load per gear width *F*_w_ is calculated as:

(2) $$F_{w}=\frac{F_{T}\;}{b}$$

where *F*_T_ is tangential load, acting on the gear’s reference diameter *d*, and *b* is the gear’s face width with unit [Ν/mm]. The relationship between values of torque, load per gear width, and root bending stress can be found in Table 6.

The experimental fatigue data were fitted using the Basquin power law, which is widely accepted as a model for the stress versus number of cycles relationship [34]:

(3) $$\Delta \sigma =C\times N_{f}^{b},\;\;\Delta \sigma =\sigma_{F}\;\mathrm{at}\;R=0$$

(4) $$\sigma_{F}=C\times N_{f}^{b}$$

where Δσ is the stress range from minimum to maximum, $\sigma_{F}$ is the maximum tooth bending stress, *N*_f_ is number of cycles to failure, and C and b are fitting coefficients. Tests for the T300-ET455 material were also conducted in the high-cycle regime, subjecting it to up to 7 × 10^7^ cycles. However, a transition point was observed for the T300-ET455 material, where the slope of the curve shifted downward (Figure 18). To account for this transition, experimental data were fitted separately for cycles up to 1.2 × 10^7^ (low-cycle fatigue) and cycles between 1.2 × 10^7^ and 7 × 10^7^ (high-cycle fatigue). From the fitted data, we can see fairly good agreement for the fitted coefficients, which are all higher than 0.9 except for the second part of the T300-ET445 curve, where the R-square of 0.86 indicates slightly worse fitting due to the scatter of fatigue data, which is typical behaviour of composite materials [35]. The fitted coefficients C and b and R-square values are given in Table 7.

The results of the gear fatigue testing indicate that the T700S-DT120 material with 18 layers of 93 g/m² and toughened resin exhibits superior durability characteristics. On average, this material shows a 12% increase in peak load capacity and 45-times higher fatigue endurance when compared to gears fabricated from T300-ET445. The fatigue life of T300-LG900 UG with thinner 93 g/m² spread fabrics and resin infusion and wet laying techniques is slightly inferior when compared to gears fabricated from T300-ET445 laminate.

In the tests, we observed a distinct limit value of the maximum torque that can be applied without immediate failure. Once the torque limit is exceeded, the material becomes overstressed, leading to a quick failure shortly after the start of the test. This rapid failure indicates the involvement of static failure mechanisms. The torque limit is significantly higher in the T700S-DT120, at 0.9 Nm, compared to the T300-ET445 and T300-LG900UV materials which both had a torque limit of 0.8 Nm.

For comparison, a PEEK fatigue curve from the research of Zorko et al. [36], which was conducted on the same testing platform, is also plotted in Figure 18, demonstrating that the PEEK material exhibits inferior durability characteristics when compared to the evaluated CFRP gears. The fatigue results of a study by Kurokawa et al. [11] were plotted for comparison as well. The fatigue results of PEEK with the addition of 15% short carbon fibres showed a performance that was even worse than pure PEEK gears. It appears that the short carbon fibres in that case probably acted as stress raisers, promoting fatigue cracks and reducing the lifetime.

Based on the experimental data obtained from the gear fatigue testing, several key findings can be deduced. First, the use of prepreg with incorporated toughened resin has been found to significantly enhance the durability of gears. This improvement can be attributed to the toughener’s ability to increase the resistance to crack initiation and propagation in the resin matrix during fatigue. Second, the use of thinner plies and a higher number of plies for the same thickness has been found to improve the fatigue life of the gears. There are several studies which state that thin plies can substantially enhance the strength and design flexibility of the composites [37,38]. This can be explained by the fact that thinner plies allow for a more homogeneous distribution of stress throughout the gear. Additionally, a higher number of plies results in a more complex load-sharing network, further reducing the likelihood of failure.

### 3.4. Analysis of Backscatter SEM and EDS

The combination of backscattered scanning electron microscopy (BS-SEM) and energy-dispersive X-ray spectroscopy (EDS) was used to identify the composition and distribution of elements in gears after meshing.

**Figure 19:**
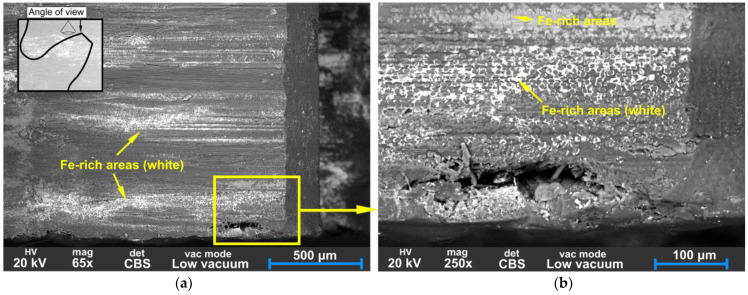
SEM backscatter images of the worn surface of CFRP gear (**a**) flank surface at the top of the tooth, 65× magnification; (**b**) detail of the crack at the edge.

In Figure 19, the BS-SEM of a tooth is shown from a top-view angle. On the worn surface, shallow peaks are visible along the sliding direction; the tops of these peaks show white areas that indicate a more Fe-rich conductive material. The rough asperities of the steel pinion can cause abrasive wear and, later, the debris from both CFRP and metal can contribute to three-body abrasion and increase the wear rate. The typical edge delamination is visible in Figure 19b. The outer surfaces of the CFRP composite material are susceptible to delamination failure due to lower adhesion between the composite plies at the edge. Typically, when two bodies are in contact, the non-restrained edge deforms more than the interior of the material, which is constrained by the surrounding material [25].

**Figure 20:**
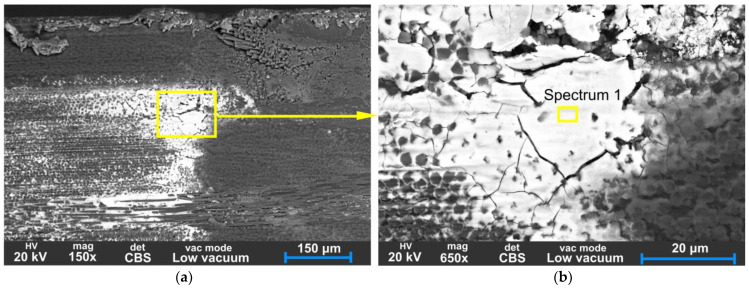
SEM backscatter images of the worn surface of CFRP gear (**a**) flank surface at the top of the tooth with the adhered layer of metal material from the steel pinion; (**b**) detail of cracks around the layer of iron.

**Figure 21:**
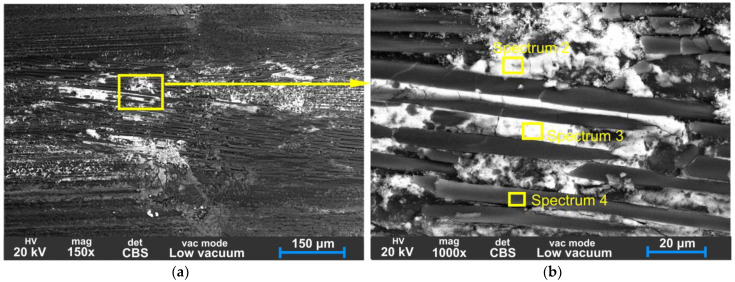
SEM backscatter images of the worn surface of CFRP gear (**a**) flank surface at the top of the tooth, 100× magnification; (**b**) detail of microcracks between fibres and matrix.

Figure 20 displays evidence of material transfer from the steel pinion onto the CFRP tooth surface. This is an example of adhesive wear, characterised by material transfer from one surface to another. As visible in Figure 21, the matrix has been crushed and separated from the fibres in the sliding direction, resulting in metal debris accumulation in cavities. During wear, continuously formed broken fibre and matrix particles act as an abrasive which causes three-body abrasion. 

**Figure 22:**
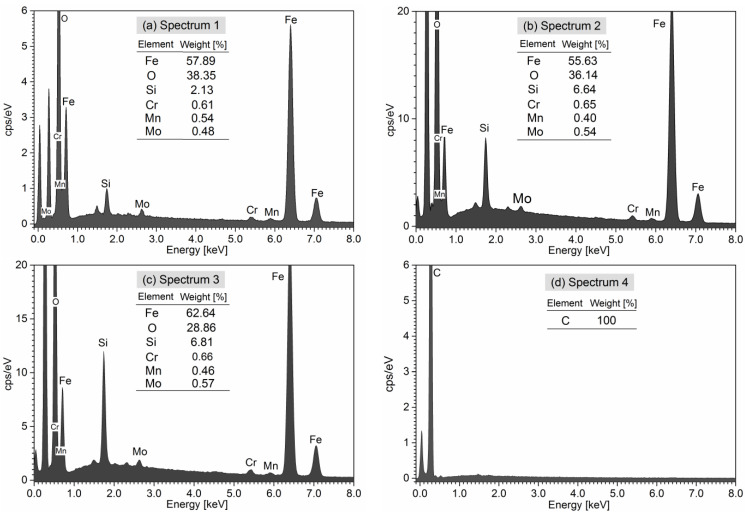
EDS analysis Spectra 1–4 representing worn surfaces at different points as presented in Figure 20 and Figure 21.

Material transfer from steel pinion onto the CFRP tooth was confirmed by EDS analysis of Spectrum 1 shown in Figure 22a, indicating the high Fe content and other metal elements which are present in the 42CrMo4 steel driving gear.

The BS-SEM images from Figure 19, Figure 20 and Figure 21 show white areas on the surface of the CFRP gear teeth, indicating the coating of adhesive debris composed mainly of Fe, Si, Cr, Mo, and Mn, which are components of the 42CrMo4 steel pinion. The EDS analyses of Spectra 1–4 are presented in Figure 22. Chemical analysis of Spectra 1–3, given in Figure 22a–c, reveal similar compositions with iron content range 55.6–62.6%, oxygen 28.8–38.3, silicon 2.1–6.8%, chromium 0.62–0.66%, manganese 0.4–0.64%, and molybdenum 0.48–0.57%. The metal chemical elements, found on the surface of the CFRP, are the elements of the 42CrMo4 steel drive gear. The 42CrMo4 steel (Wr. No. 1.7227) nominally consists of 0.38–0.45% carbon, 0.4% silicon, 0.6–0.9 of manganese, 0.9–1.2 of chromium, and 0.15–0.3 of molybdenum. This implies that the steel gear was also wearing during the meshing, transferring the metal material into the surface of the CFRP gears.

In Spectrum 4, shown in Figure 22d, only the presence of carbon was found on the carbon fibre surface. High oxygen content, detected in Spectra 1–3, is likely from the epoxy matrix or from iron oxide (FeO) formed as a result of oxidative wear on the steel drive gear. The oxides can also result from galvanic corrosion wear caused by the presence of electrolytes due to humidity.

## 4. Conclusions

The analysis of the lifespan of gears made of laminated composites compared to plastic and metal gears has shown that they have the potential to be used for high-strength applications. However, their limited utilisation in industry is attributed to the high costs associated with this technology. Despite this, as laminated composite gears have been found to have superior performance over conventional plastic injection-moulded gears with the addition of short fibres, it is likely that this trend will change with advancements in technology and cost reductions. The emergence of 3D-printed continuous fibre technology presents a significant opportunity for the efficient and cost-effective production of high-quality gears. The findings of this study allow the following conclusions to be drawn:

- The use of T700S-DT120 prepreg with incorporated toughened resin has been found to significantly enhance the durability of gears over T300-ET445 and T300-LG-900UV. This can be attributed to the higher strength fibres and toughener’s ability to improve the resistance to crack initiation and propagation during fatigue and wear. Second, the use of thinner plies results in a more complex load-sharing network, further reducing the likelihood of failure.
- The results of experimental wear study indicate nearly linear correlation between wear volume, wear pitch distance, and duration of testing. Furthermore, a consistent increase in the slope of the linear function was observed with increasing loading torque.
- The tooth profile was altered by wear, particularly in the root area, where the shape of the profile changed from an outer arched form to an inner arched shape. Numerical analysis showed that the worn out profile of CFRP gears led to a double contact with the steel pinion, heightened backlash, and elevated transmission error, negatively impacting the performance of the gears, and causing an increase in noise and vibrations and decreasing the efficiency.
- BS-SEM and EDS analysis revealed that the degradation of CFRP is due to severe adhesion and three-body wear, resulting in edge delamination caused by limited adhesion strength between the matrix and fibres. Fatigue in CFRP is typically caused by cyclic stress–strain loading, which leads to the formation of microcracks in the matrix material and eventual delamination or fibre breakage.

These findings provide valuable insights for the optimisation of CFRP gears in terms of material selection, resin systems, and layup strategies. Carbon fibre reinforced plastic (CFRP) gears have the potential to be used as a lightweight and durable alternative to traditional steel gears in various applications.

Nevertheless, when meshed with steel pinion gears, the wear of CFRP gears is a concern that needs to be addressed in the future to improve the wear resistance. This may involve testing a range of different fibres and abrasion-resistant matrix materials, as well as varying the layup and manufacturing processes to determine the best combination for reducing wear.

## 5. Patents

The patent resulting from the work reported in this manuscript is as follows:

ZORKO, Damijan, ČERNE, Borut, BERGANT, Zoran. Zobnik iz kompozitnega materiala s kontinuirnimi vlakni: patent SI 26212 A, 30 December 2022. Ljubljana: Urad Republike Slovenije za intelektualno lastnino, 2022. 10 f., 3 f. pril., ilustr. http://www3.uil-sipo.si/PublicationServer/documentpdf.jsp?iDocId=52003&iepatch=.pdf, accessed on 30 December 2022.

## Figures and Tables

**Table 1:** Fibre type and main datasheet parameters of fabrics used in panels.

#	Fibre Type	Fabrics Designation	Tex	Areal Density [g/m^2^]	Ply Thickness [mm]	Tensile Strength [MPa]	Elongation [%]	Elastic Modulus [GPa]
1	T300	CC202	200 (3K)	200	0.26	3530	1.5	230
2	T700S	66090P	67 (1K)	93	0.11	4900	2.1	230
3	T300	Style 469 spread	67 (1K)	93	0.11	3530	1.5	230

**Table 2:** Datasheet parameters of epoxy resins used in panels.

Resin	Cure Temperature [°C]	Glass Transition *T*_g_ [°C]	Tensile Strength [MPa]	Elastic Modulus E [GPa]	Elongation [%]
ET445 ^1^	80–150	135	-	-	-
DT120, toughened ^1^	100–135	110–125	-	-	-
LG 900 UV	80–110	60–90	82	3.4	6–6.5

^1^ Mechanical data for resin in prepreg are not available.

**Table 3:** Laminated panels.

Panel No.	Designation Fibre Resin	Plate Thickness [mm]	Stacking Sequence	Total Number of Plies	Manufacturing Process
1	T300-ET445	2.05	[(45/0)_2_]_s_	8	Prepreg-AC ^1^
2	T700S-DT120	2.02	[(0/22.5/45/67.5)_2_]_s_	18	Prepreg-AC ^1^
3	T300-LG 900 UV	2.02	[(0/22.5/45/67.5)_2_]_s_	18	VI-AC ^2^

^1^ Prepreg-AC: prepreg vacuum bagging and autoclave. ^2^ VI-AC: vacuum infusion and autoclave.

**Table 4:** Geometrical parameters of the test gears.

Parameter	Symbol	Value
Profile	-	Involute ISO 53A
Module	*m* [mm]	1
Number of teeth	Z	20
Pressure angle	α [°]	20
Profile shift coefficient—pinion	x_1_	0
Profile shift coefficient—gear	x_2_	0
Transverse contact ratio	ε_α_	1.557

**Table 5:** Mechanical properties of ET 445 CIT prepreg with [(45/0)_2_]_s_ configuration and 48% of fibre volume ratio.

Mechanical Property	Value	Description
*E*_1_*= E*_2_ [GPa]	46.9	Axial, transverse in-plane stiffness
*E*_3_ [GPa]	6	Transverse out-of-plane stiffness
$n_{12}$	0.08	Major Poisson ratio
$n_{23}=n_{13}$	0.3	Minor Poisson ratio
$G_{12}\;$[GPa]	17	In-plane shear stiffness
$G_{23}=G_{13}$ [GPa]	3.37	Out-of-plane shear stiffness
$X_{1}^{T}=X_{2}^{T}\;$[MPa]	292	Axial/y-transverse tensile strength
$X_{3}^{T}\;$[MPa]	50	Transverse z-tensile strength
$X_{1}^{C}=X_{2}^{C}\;$[MPa]	−217	Axial/transverse compression strength
$X_{3}^{C}\;$[Mpa]	−80	Transverse compressive strength
$e_{1}^{T}=e_{2}^{T}$	0.0095	Axial/y-transverse tensile strain limit
$e_{3}^{T}$	0.003	Transverse tensile z-strain limit
$e_{1}^{C}=e_{2}^{C}$	−0.011	Axial/y-transverse compressive strain limit
$e_{3}^{C}$	−0.011	Transverse compressive z-strain limit
$g_{12}=g_{13}$	0.019	*xy* and *xz* shear strain limit
$g_{23}$	0.014	*yz* shear strain limit
*T*_g_ [°C]	149	Glass transition temperature
*ρ* [g/cm^3^]	1.464	Density

**Table 6:** Relations between torque, load per gear width, and root bending stress VDI.

Torque [Nm]	Load per Gear Width [N/mm]	Root Bending Stress VDI [ΜPa]
0.4	20	66
0.5	25	83
0.6	30	100
0.7	35	119
0.8	40	133
0.9	45	150

**Table 7:** Coefficients C and b for regression-fitted Basquin equation to experimental data.

Material	C	b	R^2^
T700S-DT120, 93 g/m^2^	224.7	−0.033	0.94
T300-ET445, 200 g/m^2^—up to 1.2 × 10^7^	199.6	−0.034	0.92
T300-ET445, 200 g/m^2^—from 1 × 2 × 10^7^ to 7 × 10^7^	14,220	−0.294	0.86
T300-LG900 UV, 93 g/m^2^	189.1	−0.031	0.91

## Data Availability

Data are contained within the article.
